# 

**Published:** 1920-11-20

**Authors:** 


### Matrons' and Sisters' Appointments.

Ashburton and Buckfastleigh Cottage Hospital.—Miss Florence L. Valentine has been appointed matron. She was trained at the Royal Albert Hospital, Devonport, and has since been ward and theatre sister at Queen's Hospital, Bethnal Green, theatre sister at the Royal Victoria and West Hants Hospital, Boscombe, Bournemouth, and theatre and ward sister at No. 1 Military Hospital, Exeter.

Cottage Hospital, Bingley.—Miss M. J. Runell has been appointed matron. She was trained at East Suffolk Hospital, Ipswich. She has since been staff nurse and acting matron at the Cottage Hospital, Bingley, sister at the Military Hospital, Seaford, night sister at Essex County Hospital, Colchester, and night sister at the Children's Hospital, Bradford.

Crumpsall Infirmary, Manchester.—Miss Harriet Augusta Head and Miss Nellie Tranter have been appointed night superintendents. They were both trained at Crumpsall, both hold the certificate of the Central Midwives Board and are members of the College of Nursing. Miss Head has been ward sister and *x*-ray and assistant operating-theatre sister at Crumpsall. Miss Tranter has also had mental training and has been ward sister and midwife of the venereal clinic at Crumpsall.

East Lancashire Tuberculosis Colony, Barrowmore, Chester.—Miss F. Helena Yoxall has been appointed matron. She was trained at the North Staffordshire Infirmary, Stoke-on-Trent, and has since been matron of Paddock Auxiliary Hospital, Oswaldtwistle, and of Porthcawl Convalescent Home, Glamorgan.

Ethel Hedley Hospital for Children, Windermere.—Miss Louisa Poole has been appointed matron. She was trained at Camberwell Infirmary and at Southampton (in fever work). She has since been sister at Broseley Hospital, Salop, at Bournemouth Sanitary Hospital, at Coventry City Hospital; sister-in-charge of the Auxiliary Military Hospital, Breeze Hill, Bootle; and ward sister at Leasowe Hospital for Children, Cheshire, where she was also housekeeping sister.

### Personalia.

Mr. A. Stanley Brunt, Secretary-Superintendent of the North Ormesby Hospital, Middlesbrough, has been appointed General Superintendent and Secretary of the Royal Albert Edward Infirmary, Wigan. Mr. Brunt has held the appointment at Middlesbrough since April 1916, and a record of his valuable services to the North Ormesby Hospital has been placed upon the Minutes of the institution. Prior to going to Middlesbrough Mr. Brunt was for six and a-half years chief assistant to the General Superintendent Secretary of the Devonshire Hospital, Buxton. He has also had other hospital experience.

Mr. Hurd R. Gruffydd, until recently acting assistant secretary at the Great Northern Central Hospital, Holloway, has been appointed, out of a large number of candidates, to the secretaryship of the County and County Borough Infirmary, Londonderry.

We are informed that the advertisement inviting applications for two positions in the Lunacy Department of Western Australia is withdrawn for the present.

